# Wilm's Tumor-1 Protein Levels in Urinary Exosomes from Diabetic Patients with or without Proteinuria

**DOI:** 10.1371/journal.pone.0060177

**Published:** 2013-03-27

**Authors:** Anuradha Kalani, Aradhana Mohan, Madan M. Godbole, Eesh Bhatia, Amit Gupta, Raj Kumar Sharma, Swasti Tiwari

**Affiliations:** 1 Department of Molecular Medicine & Biotechnology, Sanjay Gandhi Postgraduate Institute of Medical Sciences, Lucknow, Uttar Pradesh, India; 2 Department of Endocrinology, Sanjay Gandhi Postgraduate Institute of Medical Sciences, Lucknow, Uttar Pradesh, India; 3 Department of Nephrology, Sanjay Gandhi Postgraduate Institute of Medical Sciences, Lucknow, Uttar Pradesh, India; Fondazione IRCCS Ospedale Maggiore Policlinico & Fondazione D'Amico per la Ricerca sulle Malattie Renali, Italy

## Abstract

**Background:**

Podocyte injury is an early feature of diabetic nephropathy (DN). Recently, urinary exosomal Wilm's tumor-1 protein (WT1), shed by renal epithelial cells, has been proposed as a novel biomarker for podocyte injury. However, its usefulness as biomarker for early diabetic nephropathy has not been verified yet. We investigated urinary exosomal WT1 in type-1 diabetic patients to confirm its role as a non-invasive biomarker for predicting early renal function decline.

**Methods:**

The expression of WT1 protein in urinary exosomes from spot urine samples of type-1 diabetes mellitus patients (n = 48) and healthy controls (n = 25) were analyzed. Patients were divided based on their urinary albumin excretion, ACR (mg/g creatinine) into non- proteinuria group (ACR<30 mg/g, n = 30) and proteinuria group (ACR>30 mg/g, n = 18). Regression analysis was used to assess the association between urinary exosomal levels of WT1 with parameters for renal function. Receiver Operating Characteristic (ROC) curve analysis was used to determine the diagnostic performance of exosomal WT-1.

**Results:**

WT1 protein was detected in 33 out of 48 diabetic patients and in only 1 healthy control. The levels of urinary exosomal WT1 protein is significantly higher (p = 0.001) in patients with proteinuria than in those without proteinuria. In addition, all the patients with proteinuria but only half of the patients without proteinuria were positive for exosomal WT1. We found that the level of exosomal WT1 were associated with a significant increase in urine protein-to-creatinine ratio, albumin-to-creatinine ratio, and serum creatinine as well as a decline in eGFR. Furthermore, patients exhibiting WT1-positive urinary exosomes had decreased renal function compared to WT1-negative patients. ROC analysis shows that WT-1 effectively predict GFR<60 ml. min-1/1.73 m^2^.

**Conclusion:**

The predominant presence of WT1 protein in urinary exosomes of diabetic patients and increase in its expression level with decline in renal function suggest that it could be useful as early non-invasive marker for diabetic nephropathy.

## Introduction

Diabetic nephropathy (DN) is a major cause of end stage renal disease affecting millions of people worldwide [1]. DN is characterized by an initial period of glomerular hyperfiltration, associated with progressively increasing proteinuria [2]. The onset and course of diabetic nephropathy can be ameliorated to a very significant degree by several interventions, if instituted at a point very early in the course of the development of this complication. This advocates an urgent need for early detection of nephropathy. Albuminuria is commonly used as a non-invasive marker of renal injury. Although its presence is appropriate in patients with advanced diabetic nephropathy, it has limited ability to predict the earliest stages of DN [3]. Furthermore, it is not specific for diabetic nephropathy and is highly variable within an individual [4]. In addition, the onset of impaired renal function in the absence of overt albuminuria has been reported in almost one-half of a cohort of type 1 diabetic patients [5] indicating also a lack of sensitivity.

Podocyte injury starts and contributes to deterioration of kidney function in patients with Diabetic nephropathy [6]. Using murine type 1 and type 2 diabetic models, glucose-induced podocyte apoptosis/depletion has been suggested as novel early pathomechanism(s) leading to diabetic nephropathy [7]. Furthermore, a recent transcriptome analysis of human diabetic kidney biopsy strongly highlighted the role of podocyte loss in diabetic nephropathy [8]. This has led to the emergence of methods for the quantitation of podocyte damage in the urinary sediment [9]–[11]. However, quantitation of damaged podocytes in urine is not only difficult but may also not provide early detection of renal injury [12], [13]. Recent detection of Wilm's Tumor 1 protein (WT1), a podocyte marker and a transcription factor, in urinary exosomes may surmount this shortcoming [13], [14]. WT1 is required for podocyte maturation and often used as a molecular marker for differentiated podocytes [15], [16].

Exosomes are small (<100 nm) vesicles that originate from fusion of internal vesicles of multivesicular bodies to plasma membrane. Urinary exosomes are secreted into urine from renal epithelial cells, they are known to contain membrane as well as cytosolic proteins, which have characteristics of all renal tubule epithelial cells including podocytes. Among the known 1,132 proteins found in urinary exosomes, about 34 have been implicated in various kidney diseases such as autosomal dominant polycystic kidney disease type 1, autosomal dominant and recessive nephrogenic diabetes, and Gitelman's syndrome etc. Thus examination of urinary exosomes could lead to the discovery of new non-invasive site-specific biomarkers for kidney disease [17].

WT1 is a zinc-finger transcription factor that plays an important role in podocyte maturation. Transcription factors are upregulated very early and facilitate the recruitment of several genes in response to renal injury. Thus WT-1 could hypothetically serve as biomarker for early renal injury. Therefore, we analyzed the expression of WT1 and TSG101 (exosome marker) in the urinary exosomes obtained from 48 type-1 diabetic patients and 25 non-diabetic healthy controls and assessed the relationship between urinary exosomal WT1 expression with biochemical parameters for renal function including urinary protein excretion, estimated GFR and serum creatinine.

## Results

### Clinical Characteristics of subjects enrolled in the study

Diabetic patients enrolled in our study had HbA1C less than 10% (normal range 4–6%). Proteinuria group had significantly higher HbA1C (%) relative to Non-Proteinuria group (9.5±1.7 versus 7.9±1.3, p = 0.02). Table 1 provides other clinical parameters of the subjects enrolled in our study. No significant differences were observed in the mean body mass index, age and sex ratio among the three groups. Proteinuria group had significantly raised serum creatinine (p = 0.0007) relative to Non-Proteinuria group and healthy controls.

**Table 1:** Clinical characteristics of the subjects enrolled in the study.

Indices	Healthy controls	Type 1 diabetic patients without proteinuria	Type 1 diabetic patients with proteinuria
N	25	30	18
Sex (male/female)	19/6	22/8	14/4
Age (years)	29±14	33±13	35±15
Serum creatinine (mg/dl)	0.80±0.15	0.91±0.23	1.65±0.86*
Urinary albumin to creatinine ratio (mg/g)	12.5±14.6	12.4±8.6	210.4±141.2*

All data presented as mean ± standard deviation of the means, random variable were analyzed through two-tailed student t-test while categorized variables were evaluated using chi-square test. A value of p<0.05 was considered significant.

* indicate significant difference relative to protienuria group. ACR; Urine Albumin-to-creatinine ratio.

### Predominant expression of WT1 protein in Urinary exosomes of diabetic patients compared to healthy controls

**Figure 1:**
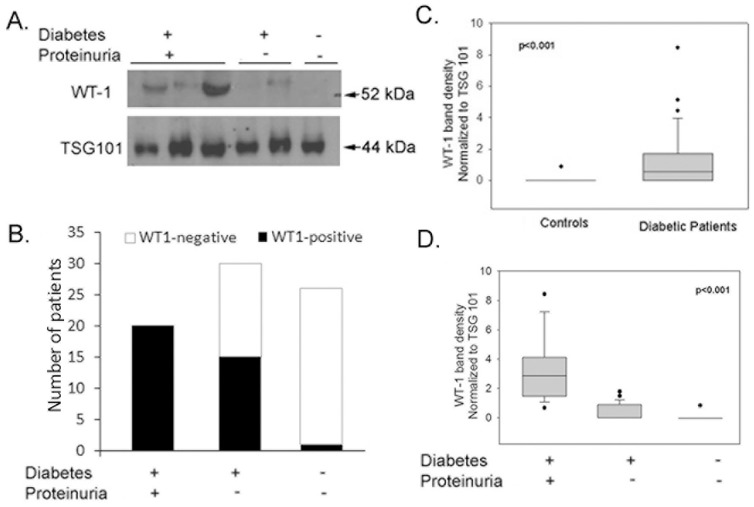
Detection of WT1 protein in urinary exosomes of diabetic patients with or without proteinuria. A) Representative immunoblots for WT1 and TSG101 proteins in urinary exosome samples from, type 1 diabetic patients with or without proteinuria and healthy controls. Exosomal protein obtained from same urine volume was loaded for all the samples. B) Frequency of WT1 expression in urinary exosomes from diabetic patients with or without proteinuria and healthy controls. All subjects were positive for TSG101 protein, an exosomal marker (data not shown). Densitometry analysis of WT1 bands in: C) Type-1 diabetic patients, using Mann-Whitney U test, and D) Proteinuria and Non-Proteinuria groups, using ANOVA rank test. The boxes indicate median and 25th and 75th percentiles; Outliers are indicated by closed dots. p<0.05 was considered significant.

Exosomal protein isolated from equal volume of spot urine samples were subjected to immunoblotting using antibody against WT1 and TSG101 protein. In addition to being an exosomal marker protein, TSG101 can also be used for the normalization of urinary exosomal protein [18]. Immunoblotting revealed a band for TSG101 protein in all subjects enrolled in the study indicating successful isolation of exosomes from all urine samples. The expression of WT1 was found in ∼69% of the diabetic patients (33 in 48) as compared to 4% in age matched controls i.e. only 1 out of 25 (Figure 1B).

### Higher expression of WT1 protein levels in urinary exosomes of Proteinuria group compared to Non-Proteinuria group and healthy controls

Predominant detection of WT1 in diabetic patients and its almost complete absence in age matched non-diabetic controls may indicate early hyperglycemia-induced podocyte injury/remodeling in diabetic patients. Therefore, we divided diabetic population based on urine albumin-to-creatinine ratio (ACR) into Proteinuria (ACR>30 mg/g creatinine) and Non-Proteinuria (ACR<30 mg/g creatinine) groups. WT1 was detected in all the patients in Proteinuria group (100%). Interestingly, 50% of diabetic patients without proteinuria (15 out of 30) were detected positive for WT1 (Figure 1B). Furthermore, Proteinuria group had significantly higher levels of WT1 compared to Non-Proteinuria and control groups (p<0.001, Figure 1D).

### Expression of WT1 protein in Urinary exosomes of Diabetic patient's associates with increase in urine protein excretion

**Figure 2:**
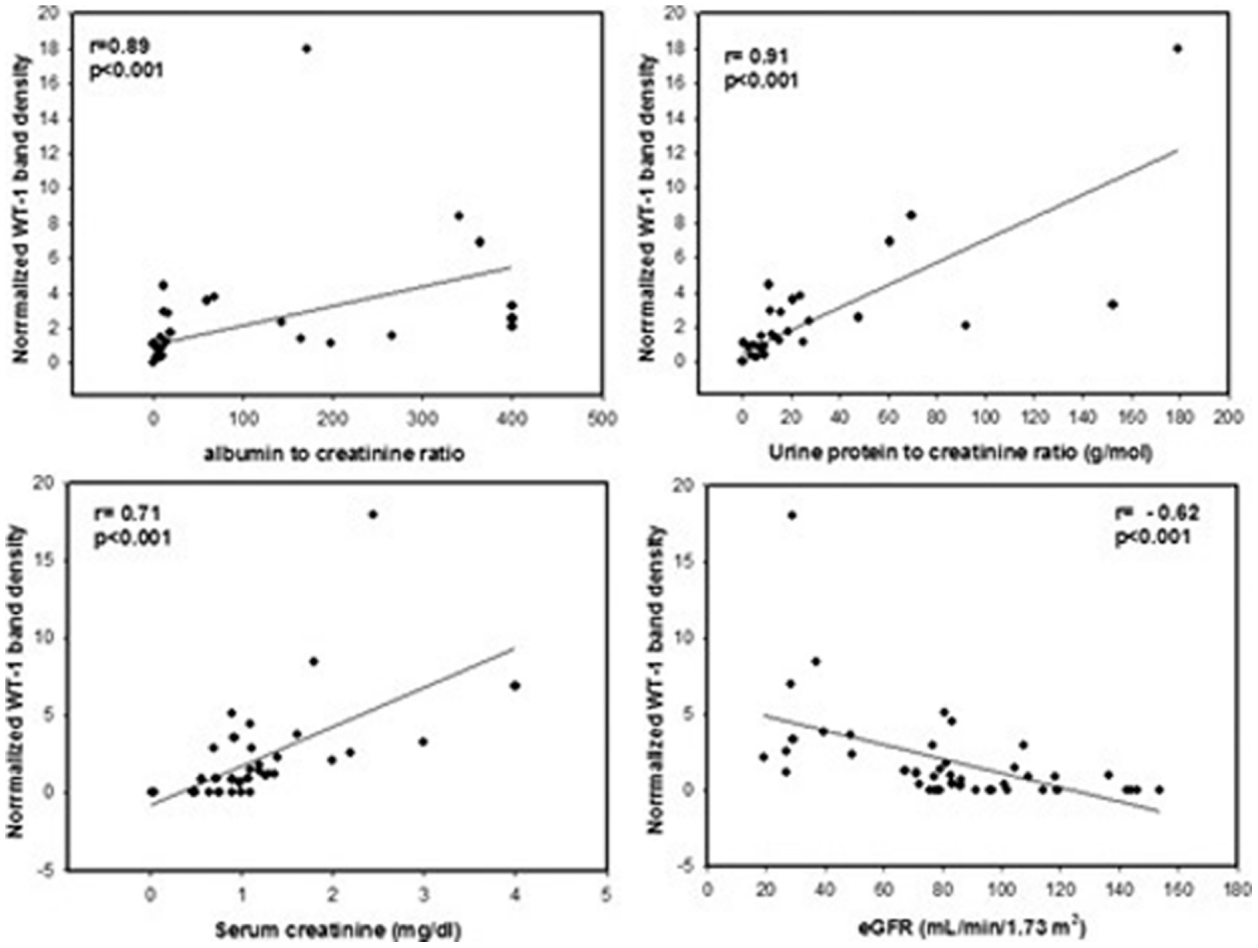
Relationship between urinary exosomal WT1 expression and parameters for renal function. Correlation of WT1 band density (normalized to TSG101 band density) with albumin to creatinine ratio (ACR); urine protein-to-creatinine ratio; serum creatinine; and estimated GFR in type-1 diabetic patients. Spearman rank-order correlation coefficient ‘r’ is given with its respective P-value, p<0.05 was considered significant.

Higher levels of WT1 observed in Proteinuria group led us to determine its relation with urine protein excretion in diabetic patients. Using regression analysis we found that WT1 protein band density associated with increase in urine protein excretion as indicated by its significant association with ACR (r = 0.89, p<0.001) and also with protein-to-creatinine ratio, UPC (r = 0.91, p<0.001) in type-1 diabetic patients (Figure 2). In addition, a significant association of WT1 levels was also observed with rise in serum creatinine (r = 0.71, p<0.001) and fall in eGFR (r = −0.62, p<0.001). These results suggest that increase in WT1 levels in urinary exosomes relates to decline in renal function in diabetic patients.

### WT1 positive patients exhibit decline in renal function parameters compared to WT1 negative subjects

**Figure 3:**
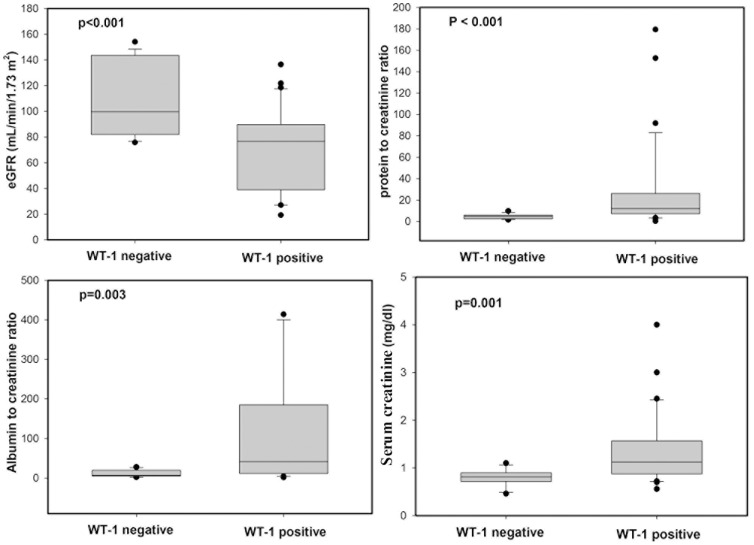
Comparison of renal function parameters between WT1 positive and WT1 negative diabetic subjects. Box plots comparing; A) Estimated GFR; B) Urine protein-to- creatinine ratio; C) Urine albumin-to-creatinine ratio; and D) serum Creatinine levels between WT1 positive and WT1 negative diabetic patients. The boxes indicate median and 25th and 75th percentiles; Outliers are indicated by closed dots. Data were compared by the Mann-Whitney U test. p<0.05 was considered significant.

In order to test if urinary exosomal WT1 could be used as a biomarker of early renal function decline in diabetic patients, we compared different parameters of kidney function in WT1 positive and WT1 negative patients. WT1 positive patients had significantly lower eGFR (p<0.001) relative to WT1 negative patients. Furthermore, significantly higher urine protein excretion (p<0.001 for UPC and p = 0.003 for ACR) and raised serum creatinine (p = 0.001) was found in WT1 positive patients compared to WT1 negative patients (Figure 3). Thus, WT1 in urinary exosome may be indicative of early decline in renal function.

### Diagnostic power of urinary exosomal WT-1 as a predictor of GFR<60 ml. min^−1^/1.73 m^2^

**Figure 4:**
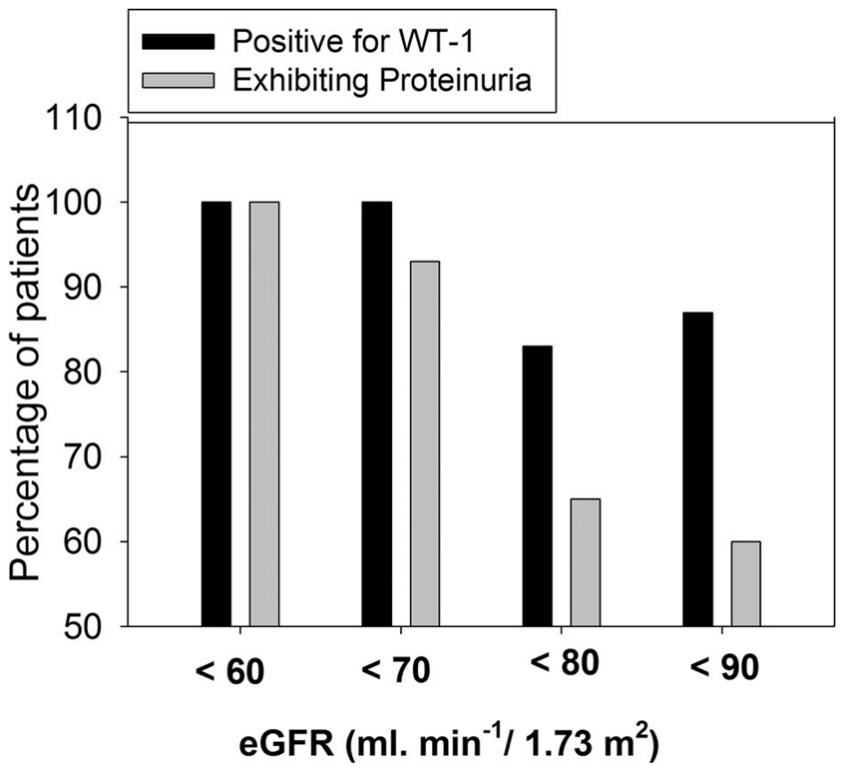
Comparison of WT-1 expression and presence of proteinuria (ACR) in diabetic patients at various eGFR cutoffs. Bar graph showing percentage of patents detected with proteinuria or WT1 expression in urinary exosomes at various cutoff values of eGFR between 60–90 ml. min^−1^/1.73 m^2^). WT-1 expression was detected in higher percentage of patients at earlier fall in GFR (eGFR<70/80/90 ml. min^−1^/1.73 m^2^).

ROC Receiver-operator characteristics (ROC) curve was calculated to assess the diagnostic power of urinary exosomal WT-1 as a predictor of lower GFR in terms of AUCs. Table 2 demonstrates that WT-1 effectively predict GFR<60 ml. min^−1^/1.73 m^2^ with an AUC of 0.92. WT-1 protein levels with a cut-off value of 1.9 provides the optimal sensitivity (88.6%) and specificity (100%) to predict GFR<60 ml. min^−1^/1.73 m^2^. Moreover, the cut-off value of 6.0 predicts with a sensitivity of 100% (95% CI: 90 to 100%). In addition, at eGFR<70/80/90 ml. min-1/1.73 m^2^, higher percentage of normoalbuminuric patients were found positive for WT-1 compared to presence of proteinuria in these patients (Figure 4), suggesting that WT-1 may effectively predict early fall in GFR.

**Table 2:** Urinary exosomal WT-1 and albumin-to-creatinine ratio as a predictor of GFR<60 ml. min^−1^/1.73 m^2^.

Test Variables	ACR	WT-1
ROC Curve Area	0.95	0.92
Standard Error	0.03	0.04
95% Confidence Interval	0.89To 1.01	0.83To 0.99
P Value	<0.0001	<0.0001
Cut-off values for optimal sensitivity with 9 5% CI	51 (88.6%, CI 73.26 to 96.8) 116 (94.3%, CI 80.8 to 99.3) 157 (97.1%, CI 85.08–99.93)	1.9 (88.6%, CI 73.26 to 96.8) 3.1 (94.3%, CI 80.8 to 99.3) 4.8 (97.1%, CI 85.08–99.93)

Table indicate data obtained from Receiver-operator characteristics (ROC) curve analysis. An AUC value of 0.5 indicates no discriminative value while the maximum value for the AUC is 1.0 which indicates perfect test. The 95% Confidence Interval is the interval in which the true (population) Area under the ROC curve lies with 95% confidence.

## Discussion

In summary, we have reported for the first time that: 1- The predominant expression of WT1 protein in urinary exosome in type-1 diabetic patients compared to its' complete absence in non-diabetic age matched controls; 2- higher levels of WT1 in patients with proteinuria relative to patients without proteinuria; 3- strong association of WT1 expression in urinary exosomes with increase in urinary protein excretion and decline in eGFR; 4- patients with detectable WT1 exhibit significantly higher urinary protein excretion, raised serum creatinine and lower eGFR relative to patients without detectable WT1 in urinary exosomes. 5- WT-1 had the ability to predict GFR<60 ml. min-1/1.73 m^2^.

WT1 has been associated with podocyte malfunction and tubulointerstitial fibrosis [19]. Since the reduction in podocyte number and density has been linked to proteinuria and progression of diabetic nephropathy, WT1, as a marker to evaluate podocyte damage, has been a major focus of the diabetic nephropathy biomarker investigations. In this regard, Su et al. [16] have used WT1 as a marker to evaluate podocyte damage and showed that podocyte number and density was decreased in patients with early stage of DN which became more dramatic as proteinuria progressed. Furthermore, Kubo et al. have for the first time reported the detection of endogenous WT1 mRNA in patients with renal disease [19]. More recently, Zheng et al. have demonstrated increase levels of urinary podocyte associated mRNA levels of synaptopodin, podocalyxin, CD2-AP, a-actin4, and podocin mRNA expression with diabetic nephropathy progression [10]. These studies have undoubtedly implicated a promising technique for diagnosing kidney disease progression and therapy response; however, this technique may not determine early podocyte injury since it involves the analysis of already damaged podocyte in the urine. Furthermore, it is important to explore alternatives approaches to the expensive and invasive method of renal biopsy for an early detection of podocyte injury.

The analysis of biomarkers of renal injury in urinary exosomes represents a promising alternative approach to renal biopsy. Since exosomes are secreted into urine from renal epithelial cells including podocyte and contain membrane as well as cytosolic proteins implicated in kidney diseases [20], [21]. Furthermore, urinary exosome account for ∼3% of the total urinary proteins and exosome isolation procedures allow >30-fold enrichment of constituent proteins including intracellular proteins and transcription factors and thus can be an alternate to renal tissue biopsy [22]. Moreover, total urinary proteins consist of plasma derived and other abundant proteins that mask low but clinically important proteins. In this regards, Zhou et al failed to see the expression of WT1 in whole urine from FSGS patients [13].

Transcription factors in urinary exosomal, such as WT1, have been suggested as a new class of biomarkers for renal diseases and that it may offer insight into cellular regulatory pathways [13]. Thus in the present study we investigated urinary exosomal WT1 to confirm its role as a non-invasive biomarker for predicting early renal function decline in type-1 diabetic patients.

In agreement with our results, Zhou et al [13] reported undetectable WT1 protein in urinary exosomes from healthy controls..Moreover, Kubo et al also failed to see the expression of WT1 mRNA in urine from healthy controls [19].

Since urinary exosomes are membrane-bound vesicles, they protect nucleic acids and proteins from degradation and, thus, they are better source of biomarkers than the whole urine. This might explain the enhanced detection of WT1 that we observed in all the diabetic patients with proteinuria. Nevertheless, Kubo et al have reported WT1 mRNA in whole urine from only 40% (8 in 20) diabetes mellitus patients with proteinuria [19].

The higher levels of WT1 observed in patients with proteinuria compared to those without proteinuria suggests its association with renal injury. We hypothesize that podocyte dedifferentiation due to hyperglycemic milieu exposure may have accounted for the appearance of WT1 in urinary exosomes as observed in our study and its concomitant loss in kidney tissue as reported by others [17], [8]. In this regard loss of podocyte markers including WT1 has been reported in renal biopsy tissue in diabetic nephropathy [17]. Furthermore, podocyte-specific transcripts including WT1 were severely decreased in diabetic kidney tissue [8].

Enhanced detection (50%) and higher levels WT1 in Non-Proteinuria group compared to healthy controls might indicate very early renal injury. Detection of urinary exosomal WT1 earlier than proteinuria and glomerular histological damage, in animal model of FSGS, further support our hypothesis [13]. Furthermore, Kubo et al. (1999) reported the presence of WT1 mRNA in total urine in 2% of the diabetic patients without macro-proteinuria while it was absent in healthy controls [19]. Toxic urinary environment may have limited the detection of WT-1 mRNA in total urine in the study done by Kubo and collegues [19].

Nonetheless significantly higher urinary protein excretion, raised serum creatinine and lower eGFR in WT1 positive compared to WT1 negative patients, and strong association of WT1 levels with increased urinary protein excretion suggest the usefulness of urinary exosomal WT1 as a non-invasive biomarker in predicting early renal injury in diabetic patients. Moreover, ACR and WT-1 were found to be equivalent in predicting GFR<60 ml. min^−1^/1.73 m^2^; however, WT-1 is present in higher percentage of patients compared to proteinuria at earlier cutoff values of GFR (eGFR<70/80/90 ml. min^−1^/1.73 m^2^) (Figure 4). This in addition to significant correlation of WT-1 expression with decline in eGFR suggest that appearance of WT-1 levels in urinary exosome may indicate very early fall in GFR. However, the present set of data in our study did not answer the question whether the cases with diabetes without proteinuria who have WT-1 in their urine go on to develop proteinuria and then show loss of GFR. Nevertheless, the detection of WT1 in animal model of FSGS followed by appearance of proteinuria and glomerular histological damage, further suggest that it could be the case [13]. Moreover, the patients in the present study are being followed up to see if non-proteinuria patients with detectable WT-1 in urinary exosome go on to develop decrease in GFR.

## Materials and Methods

### Ethics Statement

All studies were approved by the Ethical Committee of Sanjay Gandhi Postgraduate Institute of Medical Sciences. Written informed consents were obtained from all subjects to use their urine for research purpose.

### Subjects and Sample collection

In this study we evaluated urinary exosomes obtained from spot urine samples of 48 patients with type-1 diabetes mellitus, who manifested normoalbuminuria (urine albumin<30 µg/mg creatinine) or microalbuminuria (urine albumin 30–300 µg/mg creatinine) and 25 healthy controls. Spot urine and blood samples were collected. Subjects with an initial HbA1c>10%, diabetic ketoacidosis, high blood pressure (>140/90 mmHg) and pregnant and/or breast-feeding women, and smokers were excluded from the study. Urine samples were screened for leucocytes; blood cells; specific gravity; bilirubin; pH; ketone; urobilinogen; and nitrite using urine test strips (Uristix, Bayer Diagnostics India Ltd, Gujarat, India). The abnormal samples were excluded. The rest of the samples were centrifuged at 2,000× g for 10 min to remove cellular debris and stored at −80°C till further processing.

### Urine and blood analysis

Spot urine and blood samples were collected from patient and control groups. Urine samples were centrifuged at 5,000 rpm for 10 min at room temperature to remove cell debris. Urine protein concentration was estimated using Bradford method (Protein assay dye reagent, Biorad, CA, USA). Urine albumin levels were estimated using immuoturbidometric assay (TURBILYTE-MA™, Tulip Diagnostics, Goa, India). Modified Jaffe's method was used for colorimetric estimation of creatinine concentration in urine and serum samples (Autospan Liquid Gold Creatinine kit, Span Diagnostics, Gujarat, India). HbA1c levels of the patients were measured in the blood (‘VARIAN™ II Hemoglobin system, Biorad, CA, USA). GFR was estimated (eGFR) by Modification of Diet in Renal Disease (MDRD) equation [23].

### Isolation of urinary exosomes and Western blotting

Urinary exosomes were isolated from 35 ml urine using a differential centrifugation method [24], [18]. Briefly, after quick thawing samples were vortexed extensively followed by centrifugations at 1,500× g and 17,000× g for 10 min each. The supernatant was subjected to ultracentrifugation (Beckman coulter LE80K, CA, USA) at 200,000× g for 2 hr at 4°C to obtain the urinary exosome pellet. The exosome pellets were re-suspended in 50 µl isolation buffer and the protein was solubilized in Laemmli sample buffer as described previously [24], [25]. Equal volume of solubilized protein (10 µl) was loaded for each sample onto 10% polyacrylamide gel. Separated proteins were transferred onto nitro-cellulose membranes and blocked with 5% non fat dry milk for 1 hr. Membranes were then incubated with primary antibodies, rabbit polyclonal WT1 (Abcam, MA, USA) and mouse monoclonal TSG101 (Abcam, MA, USA) in 1∶500 dilution overnight at 4°C. TSG101 was used for the normalization of urinary exosome [18]. After the incubation with primary antibody, membranes were washed and then incubated with horse radish peroxidase conjugated appropriate secondary antibody (1∶10,000). The antibody-antigen reactions were visualized by using chemiluminescence (GE Healthcare, NJ, USA).

### Statistical analysis

Quantitative data are expressed as mean ± Standard deviation. Differences between groups were determined by unpaired student t-test and chi square test (for parametric variables). Correlation analyses were performed by Spearman rank order correlation. Receivers operating characteristic curves were plotted using Sigma Plot 10 (Chicago, IL) to describe the ability of WT-1 as a predictor of GFR<60 ml. min^−1^/1.73 m^2^. An area under the ROC curve of 1.0 represents perfect discrimination, whereas an area of 0.5 represents chance discrimination. P values<0.05 were considered significant. All the statistical analysis was done using Sigma Stat 3.5 (Chicago, IL).
